# Immune Checkpoint Inhibitors and Allograft Rejection Risk: Emerging Evidence Regarding Their Use in Kidney Transplant Recipients

**DOI:** 10.3390/jcm14145152

**Published:** 2025-07-20

**Authors:** Muhammad Ali Khan, Munir Mehmood, Hind EL Azzazi, Samiullah Shaikh, Bhavna Bhasin-Chhabra, Prakash Gudsoorkar, Sumi Sukumaran Nair, Lavanya Kodali, Girish Mour, Sundararaman Swaminathan, Bassam G. Abu Jawdeh

**Affiliations:** 1 Division of Nephrology and Hypertension, Mayo Clinic Arizona, 5777 E. Mayo Blvd, Phoenix, AZ 85054, USA; bhasin-chhabra.bhavna@mayo.edu (B.B.-C.); nair.sumi@mayo.edu (S.S.N.); kodali.lavanya@mayo.edu (L.K.); mour.girish@mayo.edu (G.M.); swaminathan.sundararaman@mayo.edu (S.S.); 2 Medical College, The Aga Khan University, Stadium Road, Karachi 74800, Pakistan; mehmoodmunir483@gmail.com; 3 Centre Hospitalier Universitaire de Rabat-Salé, Mohammed V University, Rabat 10000, Morocco; hindelazzazi@gmail.com; 4 Internal Medicine, Liaquat University of Medical & Health Sciences, Jamshoro 76080, Pakistan; sami.shaikh581@gmail.com; 5 Division of Nephrology, University of Cincinnati, 231 Albert Sabin Way, Cincinnati, OH 45267, USA; gudsoops@ucmail.uc.edu

**Keywords:** immune checkpoint inhibitors, kidney transplantation, graft rejection, post-transplant malignancy, programmed cell death 1 receptor, programmed cell death 1 ligand 1 protein, CTLA-4 antigen

## Abstract

The indications for immune checkpoint inhibitor (ICI) use in cancer treatment continue to expand. This is attributable to their proven anticancer activity in addition to their tolerability and favorable toxicity profile as compared to conventional chemotherapeutic agents. ICIs work by blocking the inhibitory signals between tumor cells and T-cells, thereby enhancing the T-cell cytotoxic activity to inhibit tumor growth. Because of their immune-stimulating effect, ICIs are linked to adverse renal outcomes in both native and transplanted kidneys. The risk of kidney allograft rejection in the setting of ICI use has been reported to be around 40%, leading to an increased risk of graft loss. In this report, we review the literature examining outcomes in kidney transplant recipients receiving ICIs for various oncologic indications.

## 1. Introduction

Kidney transplantation (KT) is considered the best available treatment for end-stage kidney disease (ESKD), as it significantly enhances patient survival and improves quality of life compared to dialysis [1,2]. Significant advances in immunosuppression medications have been achieved over the last several decades where acute rejection rates have decreased significantly leading to improved short-term outcomes. Maintaining longer term outcomes remains challenging, however, due to ongoing risk of chronic rejection, infections, and medication side effects [3,4,5].

One concern in KT recipients is their increased risk of developing cancer compared to the general population. Various studies have consistently demonstrated incidence rates of skin cancer, lymphoproliferative disorders, and solid organ tumors significantly above the non-immunosuppressed population [6,7,8]. Skin cancer, particularly squamous cell carcinoma, is the most frequently observed malignancy attributed to cumulative immunosuppressive burden and chronic ultraviolet radiation exposure [9,10,11,12]. Moreover, the risk of lymphomas in transplanted patients over a 10-year period is 11.8-fold higher than in non-transplanted patients, with the majority occurring within the first year post-transplant [13].

Numerous factors contribute to the heightened risk of cancer development in transplant patients. Long-term immunosuppressive therapy significantly compromises the immune system’s natural ability to detect and eliminate cancerous cells, rendering transplant recipients especially susceptible to malignancies. In an immunocompetent host, early responders like natural killer T-cells (NKTs), natural killer cells (NKs), and T-cells detect stressed or mutated cells and release interferon gamma (IFN-y) [14]. This hinders tumor cell division and sparks the production of chemokines (CXCL9, CXCL10, CXCL11) that starve the lesion of new blood vessels while attracting more NK cells, macrophages, and dendritic cells [15,16]. Dendritic cells clear away tumor debris and act as a bridge between innate and adaptive immunity, activating both helper and cytotoxic T-cells to target and destroy cancer cells [17,18]. When any link in this chain is weakened by immunosuppression, the resulting decline in IFN-γ, Interleukin-12, and T-cell numbers impairs the complete elimination of tumors. Instead, residual variants persist in an equilibrium state under sub-optimal immune pressure, eventually acquiring mutations that let them escape, expand unchecked, and become clinically evident cancers [19].

Chronic oncogenic viruses including Epstein–Barr virus (EBV), high-risk human papillomavirus (HPV), and hepatitis B and C viruses increase the risk of cancer in immunocompromised hosts [20,21]. Prolonged immunosuppression allows these latent viruses to reactivate. Their oncoproteins trigger unchecked cell proliferation, disable p53/pRb tumor suppressor pathways, and induce genomic instability, thereby accelerating malignant transformation [22,23]. Once tumors emerge, they deepen immune escape by up-regulating inhibitory checkpoint proteins that blunt T-cell activity [24]. Targeting these checkpoints with Food and Drug Administration (FDA)-approved immune checkpoint inhibitors (ICIs) has revolutionized therapy for cutaneous squamous cell carcinoma, melanoma, several solid tumors, and lymphomas, malignancies to which KT recipients are particularly susceptible [25].

In KT recipients, however, the use of ICIs poses distinct challenges. Due to their immune-activating effects, transplant patients treated with ICIs face an increased risk of acute rejection and graft loss [26,27]. In this review, we systematically explore and summarize the available evidence on the effects, outcomes, and challenges associated with the use of ICIs in KT recipients.

## 2. Immune Checkpoint Inhibitor Mechanism of Action

Checkpoint proteins constitute specialized regulatory molecules expressed on T-cells, which are critical in modulating immune responses. They function as inhibitory mechanisms, analogous to “brakes,” preventing excessive T-cell activation, thereby preserving immunological homeostasis and mitigating the risk of autoimmune reactions [28]. In solid organ transplantation, the same brakes help dampen alloreactive T-cell responses and promote the accommodation of the graft.

One prominent checkpoint protein is cytotoxic T-lymphocyte-associated protein 4 (CTLA-4). Effective T-cell activation generally requires two distinct signals. The initial signal is the antigen recognition by the T-cell receptor, and the second co-stimulatory signal is from the interaction between CD28 molecules on the T-cell and CD80/CD86 molecules on antigen-presenting cells (APCs) [29,30]. CTLA-4 competes directly with CD28 for binding to CD80/CD86, thereby obstructing this essential co-stimulatory interaction [28]. Consequently, T-cells fail to achieve full activation and instead transition into a state of functional inactivity known as anergy [31,32]. Within an allograft, this pathway curtails the expansion of donor-specific naïve T-cells and aids regulatory T-cell-mediated tolerance.

Another crucial checkpoint molecule is programmed cell death protein 1 (PD-1), which is similarly expressed on T-cells. PD-1 interacts primarily with programmed cell death ligand 1 (PD-L1), a ligand frequently expressed on tumor cells and APCs. Engagement of PD-1 by PD-L1 transmits inhibitory signals to T-cells, significantly diminishing their immune effector function. This immunosuppressive interaction enables tumor cells to evade immune-mediated recognition and destruction, facilitating their survival, proliferation, and potential metastasis [33]. Although CTLA-4 and PD-1 act at different checkpoints, their combined inhibition of allo- and tumor-reactive T-cells in transplant hosts accelerates oncogenesis and complicates oncologic management in this vulnerable population.

**Figure 1:**
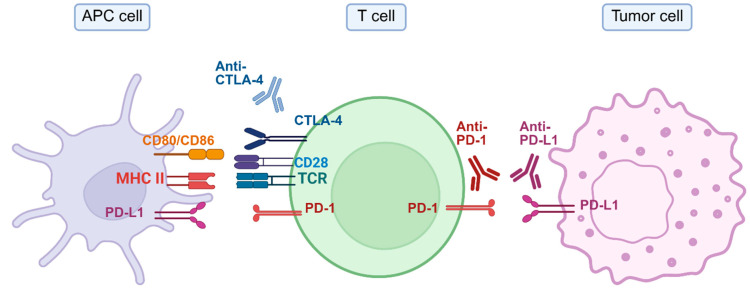
The role of checkpoint proteins in tumor immunity. This diagram illustrates the interactions between APCs, T-cells, and tumor cells, highlighting the role of immune checkpoint proteins and their therapeutic blockade. The APC expresses MHC class II, CD80/CD86, and PD-L1, which interact with TCR, CD28, CTLA-4, and PD-1 receptors on the T-cell, respectively. Normally, the engagement of CTLA-4 with CD80/CD86 inhibits T-cell activation, a process blocked by anti-CTLA-4 antibodies. In the tumor microenvironment, tumor cells express PD-L1, which binds to PD-1 receptors on T-cells, suppressing their activity and allowing for immune evasion. ICIs such as anti-PD-1 and anti-PD-L1 antibodies restore T-cell function by preventing this inhibitory interaction, thus enhancing antitumor immunity. Anti-CTLA-4: antibody against CTLA-4; Anti-PD-1: antibody against PD-1; Anti-PD-L1: antibody against PD-L1; APC: antigen-presenting cell; CD80/CD86: co-stimulatory molecules on APCs; CTLA-4: cytotoxic T-lymphocyte antigen 4; MHC II: major histocompatibility complex class II; PD-1: programmed cell death 1; PD-L1: programmed cell death ligand 1; TCR: T-cell receptor. Created in BioRender. Khan, M. (2025) https://BioRender.com/x45ra2c.

Antibodies against CTLA-4 (ipilimumab, tremelimumab) allow CD28 on naïve T-cells to bind CD80/86 again. As a result, more tumor-specific T-cells are activated, and the number of suppressive regulatory T-cells inside the tumor falls. Conversely, antibodies against PD-1 or PD-L1 (nivolumab, pembrolizumab, durvalumab) act later on effector T-cells that have reached the tumor but lost function. Blocking the PD-1 signal restores cytokine release (IFN-γ, Tumor Necrosis Factor (TNF), Interleukin 2) and preserves a pool of memory T-cells that sustains long-term tumor control [34,35,36]. Figure 1 outlines the mechanism of action of ICIs.

## 3. Overview of the Challenges Associated with Using Immune Checkpoint Inhibitors

The FDA has approved various ICIs for cancer therapy [25], including PD-1 receptor inhibitors (e.g., cemiplimab, nivolumab, and pembrolizumab), PD-L1 inhibitors (e.g., atezolizumab, avelumab, and durvalumab), and CTLA-4 inhibitors (ipilimumab and tremelimumab). ICIs have offered new hope for cancer patients, especially for those with immune-active tumors classified as “hot tumors”, cancers whose microenvironment is already inflamed, with dense CD8^+^ T-cell infiltration, high interferon-γ signaling, and abundant PD-L1 expression. In this setting, ICIs reinvigorate exhausted T-cells and can produce durable remissions [37,38,39]. The expanded use of ICIs has been associated with a range of complications, however, including immune-related adverse events (irAEs) and allograft rejection in solid organ transplant recipients. In this review, we focus on renal adverse events, particularly the risk of rejection and kidney allograft loss. A brief overview of other systemic irAEs is provided in Table 1.

### 3.1. ICI-Associated Acute Kidney Injury

ICI-associated acute kidney injury (AKI) is a term that describes AKI specifically attributed to irAEs of ICIs in the kidney. The diagnosis is usually based on clinical suspicion with or without a supportive kidney biopsy. Acute tubulointerstitial nephritis (ATIN) is the most common finding in kidney biopsies of patients with ICI-associated AKI (seen in 80–90% of cases) [53]. AKI can also be mediated by the occurrence of glomerular disorders including pauci-immune glomerulonephritis, minimal change disease, complement 3 glomerulonephritis, and IgA nephropathy [59]. ATIN, however, remains the predominant etiology for AKI with ICI use. The median time of onset is usually 16 weeks after ICI administration, though AKI can occur as early as one week or may be delayed to a year or longer after the initiation of ICI [53,60]. The risk factors for ICI-associated AKI include low GFR, prior irAEs, and proton pump inhibitor use [53]. Patients experiencing ICI-associated AKI generally have a favorable prognosis, with kidney recovery reported in 64–85% of cases [54]. Assessing renal function prior to each infusion, avoiding nephrotoxic agents, addressing hypovolemia, and assessing any reversible cause of acute kidney injury is paramount to mitigating the risk of ICI-associated AKI [61].

Corticosteroids remain the primary treatment. Among patients treated with corticosteroids, early initiation (within 3 days of ICI-associated AKI) was associated with higher odds of renal recovery compared with later initiation [53]. The recommended dosage for prednisone is 0.8–1.0 mg/kg for a duration of 6–8 weeks per recently published guidelines. Patients who relapse despite an appropriate course of steroids can be treated with an addition of a TNF alpha inhibitor, infliximab. The recommended dose of infliximab is 5 mg/kg, and this can be used as a one-time dose or can be continued monthly depending on clinical response and resolution of AKI. Mycophenolate mofetil has also been reported to be used in some cases. This guideline recommends against the use of cyclosporine, cyclophosphamide, or azathioprine for ICI-associated AKI. It is important to remember that ICI-associated AKI can also result from glomerular disorders caused by ICIs, and this requires a more nuanced approach to management [60].

### 3.2. Differentiating ICI-Associated Acute Tubulointerstitial Nephritis from Allograft Rejection

In KT recipients, distinguishing ICI-associated ATIN from rejection is challenging due to overlapping clinical features [52]. However, there are several key distinctions based on clinical presentation, laboratory findings, and histopathological features.

In both cases, marked interstitial infiltrate, consisting primarily of T-cells and monocytes, may be observed along with tubulitis. However, ICI-associated ATIN may present with other concomitant irAEs and display specific features, including granulomatous lesions (20%) and eosinophilic infiltrates (57%), which are much less likely to be found in ICI-associated acute T-cell-mediated rejection. On the other hand, intimal arteritis is very unusual in ATIN and more common in T-cell-mediated rejection [54]. Furthermore, ICI-associated ATIN usually occurs after a prolonged period of treatment with ICIs (weeks to months) as compared to ICI-associated rejection, which can cause an acute rapid decline in kidney function within days [62]. The difference is also evident from urinalysis and blood tests. The presence of eosinophils and WBC casts suggests ICI-ATIN, while the absence of these findings and the presence of C4d deposition or vascular changes may indicate rejection [52,63].

## 4. Literature Examining Immune Checkpoint Inhibitor Use in Kidney Transplant Recipients

### 4.1. Methods

While the previous sections provided an overview of ICIs, understanding their specific implications in KT recipients requires a structured analysis of the existing literature. Therefore, we conducted a narrative review to explore trends in how ICIs have been utilized in KT patients, focusing specifically on rejection rates, graft survival, and mortality outcomes reported across various study designs.

We searched PubMed, Embase, and Scopus databases from inception to January 2025, using terms related to ICIs, kidney transplant patients, and rejection. Eligible studies included clinical trials, observational studies, case series, and case reports that specifically reported outcomes in KT recipients treated with immune checkpoint inhibitors. Studies involving other types of organ transplants only, non-transplant populations, and those that were solely on animal research were excluded. Following the initial screening of titles and abstracts, full texts of relevant articles were reviewed, resulting in 33 studies included for qualitative synthesis. The extracted data encompassed patient demographics, transplant characteristics, ICIs used, rejection rates, graft survival, adverse events, and patient survival. Due to significant methodological and clinical heterogeneity, a descriptive, narrative synthesis was employed to summarize findings, identify trends, and explore clinical implications (Table 2).

### 4.2. Results

#### 4.2.1. Clinical Trials

The use of ICIs in KT recipients has been explored in recent trials, with particular attention to rejection rates, graft outcomes, and patient mortality. Treatment-related allograft loss (TRAL), i.e., graft loss that could be attributed to immunotherapy, was a major issue in Schenk et al.’s cohort, with three patients experiencing it [64]. All three of these patients experienced allograft rejection at about 6 weeks after starting nivolumab therapy and 11 weeks after starting nivolumab + ipilimumab treatment. Evidence of T-cell-mediated rejection (TCMR) was found in all three patients, with two patients also showing signs of antibody-mediated rejection (ABMR). On the other hand, the cohort of Hanna et al. examining cemiplimab in cutaneous squamous cell carcinoma fared significantly better, with no allograft rejection events observed during the study and no patients requiring hemodialysis [65]. Although these results may suggest that cemiplimab is superior to nivolumab ± ipilimumab when considering allograft rejection, no definitive conclusions can be drawn due to the limited sample size of both of these trials. These findings do highlight the importance of conducting larger-scale trials to thoroughly assess and address potential safety concerns. Interestingly, Schenk et al. also demonstrated that elevated donor-derived cell-free DNA (dd-cfDNA) levels could predict rejection earlier than serum creatinine increases, suggesting that it could potentially be a useful biomarker for monitoring patients undergoing immunotherapy [64]. Carroll et al. tested nivolumab in 17 KT recipients while maintaining baseline immunosuppression [66]. Acute rejection occurred in two patients (12%) within the first 5 weeks. The main design difference was the mandatory maintenance of baseline immunosuppression and the exclusion of recipients with high-titer donor-specific antibodies.

Adverse events were common and were graded using the National Cancer Institute Common Terminology Criteria for Adverse Events, version 5.0. Schenk et al. reported ≥ Grade 3 treatment-related adverse events in two patients (AKI and anemia) being treated with nivolumab and three patients being treated with the combination of ipilimumab and nivolumab [64]. Similarly, Hanna et al. reported that all twelve patients experienced adverse events of any grade [65]. Grade 3 events were experienced by ten (83%), and Grade 5 were experienced by three (25%) of the patients. These results once again highlight the importance of the appropriate selection of patients for ICI treatment, as the treatment regimen can be intolerable for some patients.

Mortality rates were notable in all cohorts. Schenk et al. reported four deaths: three from progressive cancer and one unrelated death due to cardiovascular complications [64]. The median overall survival in this cohort was 9.1 months (95% CI, 3.9–not estimable (NE)). Conversely, Hanna et al. reported five deaths: two attributed to progressive cancer, two unrelated to cancer (due to comorbid conditions), and one due to respiratory failure from angioedema possibly linked to everolimus and angiotensin converting enzyme inhibitor [65]. The median overall survival was significantly longer at 22.5 months (90% CI, 2.9 to 29.8). Carroll et al. reported a 53% cancer-related death rate and a median overall survival (OS) of 3.2 months (95% CI, 0.8–not reported (NR)) [64,65,66].

Among the prospective trials, Carroll et al. uniquely demonstrates that leaving carefully selected immunologically low-risk KT recipients on an established, low-dose multi-drug immunosuppressive regimen decreases the risk of ICI-associated rejection [66]. Protocols that modify or minimize immunosuppression show a spectrum of outcomes, however, from favorable to poor [64,65]. Future trials should stratify patients by baseline alloimmune risk and compare various immunosuppression strategies to define the optimal balance between cancer therapy and graft rejection.

#### 4.2.2. Retrospective Cohort Studies

Retrospective cohort studies provide varied insights into graft rejection rates, graft outcomes, mortality, and adverse events associated with ICIs in KT recipients. The interpretation of these findings, however, requires caution due to limited data, small sample size, and inconsistency across reported outcomes.

Murakami et al. highlighted substantial graft rejection rates, with 29 out of 69 KT recipients (42%) experiencing rejection after ICI initiation, leading to graft loss and dialysis dependence in 19 patients (28%) [68]. In contrast, Owoyemi et al. reported rejection in one out of seven patients (14%), occurring 21 days following cemiplimab initiation [67].

Mortality among KT recipients treated with ICIs is notably high, predominantly due to cancer progression and complications following graft rejection. Murakami et al. reported 16 deaths of the 29 patients with graft rejection (55%) [68]. Similarly, Owoyemi et al. documented a mortality rate of 57% (four out of seven patients), primarily driven by disease progression in three cases, while one death was due to severe infection [67].

Adverse events, especially graft rejection and related complications, underscore the critical role of immunosuppressive regimen adjustments. Murakami et al. identified that the use of mammalian target of rapamycin (mTOR) inhibitors was associated with a lower risk of graft rejection (*p* = 0.021) [68]. This association indicates a possible association between mTOR inhibitor use and lower rejection incidence, although this observation arises from cohort data and requires confirmation in randomized studies.

The choice and modification of immunosuppressive regimens significantly influenced graft outcomes in KT recipients treated with immune checkpoint inhibitors (ICIs). Murakami et al. reported substantial regimen adjustments prior to initiating ICIs, with 45 patients (65%) undergoing modifications [68]. The majority of these patients (62%) maintained the same number of immunosuppressive agents, whereas 35% experienced a reduction and 3% had an increase in immunosuppression. The most common adjustment involved switching from calcineurin inhibitors (CNIs) to mTOR inhibitors (15 patients), followed by the discontinuation of antimetabolites and escalation of corticosteroid dosage (14 patients each). At the initiation of ICIs, half (49%) of the patients were receiving dual-agent immunosuppression, and corticosteroids were part of the regimen in 86% of patients. Similarly, Owoyemi et al. highlighted that most patients with controlled disease were maintained on multiple immunosuppressive agents [67]. These findings emphasize the critical role of tailored immunosuppressive regimen selection and modification to enhance graft protection during ICI therapy. Although detailed cancer outcomes are beyond the scope of this review, it is briefly noted that certain cohorts demonstrated modest survival benefits associated with ICIs, particularly in cutaneous squamous cell carcinoma (cSCC), as reported by Murakami et al. (median overall survival 19.8 months vs. 10.6 months in untreated matched controls; *p* = 0.016) [68]. However, such advantages were not evident in melanoma cohorts or clearly established in Owoyemi et al.’s small patient sample [67,68].

In summary, retrospective studies highlight significant graft rejection risk, substantial mortality, and the pivotal role of tailored immunosuppressive strategies for KT recipients receiving ICIs.

#### 4.2.3. Case Series and Case Reports

##### Risk of Rejection and Graft Survival in Mono- vs. Combined Therapy

According to our included studies, ICIs including PD-1, CTLA-4, and PD-L1 inhibitors were used to treat cancers in KT recipients, either as mono- or combined therapies. PD-1 inhibitors, including nivolumab, pembrolizumab, and cemiplimab, were the most common ICIs used in our included studies (68%, 36 out of 53 patients). Cemiplimab was reported in two cases and exhibited mixed outcomes, with one patient experiencing graft rejection and another maintaining the graft [72,93]. On the other hand, nivolumab and pembrolizumab were associated with high rejection rates in several reports, resulting in a T-cell-mediated immune response and graft loss in the majority of KT recipients [74,78,79,80,82,84,87,89,90,92,94].

Similarly, in a series of seven patients, Lesouhaitier et al. reported the incidence of graft rejection following ICI administration [69]. Among the five patients receiving PD-1 inhibitors, including nivolumab and pembrolizumab, 60% (3/5) of patients experienced rejection with subsequent graft loss, while the remaining 40% (2/5) retained their grafts. In contrast, neither of the two patients treated with the CTLA-4 inhibitor, ipilimumab, and the PD-L1 inhibitor, avelumab, respectively, experienced rejection [69].

The latter observation that monotherapy with either CTLA-4 or PD-L1 inhibitors was not associated with graft rejection in Lesouhaitier et al.’s study [69] is further supported by findings from Zehou et al., which reported that among six patients receiving ICIs for metastatic melanoma, all four patients treated with ipilimumab experienced no graft rejection [70]. Similarly, Delyon et al. reported four patients, including one who received avelumab for Merkel cell carcinoma and had no graft rejection [72].

In addition to monotherapy, several studies investigated the use of combination ICI therapy in KT recipients with cancer [70,71,72,73,76,85,88,91]. One study reported the use of ipilimumab and dacarbazine, a chemotherapeutic agent, which led to T-cell-mediated rejection with graft survival [70]. Another study investigated the combination of PD-1 inhibitors, nivolumab followed by pembrolizumab, leading to T-cell-mediated rejection and graft loss [85].

The most frequently studied combination regimen included a CTLA-4 inhibitor (ipilimumab) and a PD-1 inhibitor (pembrolizumab, nivolumab, or both) [70,71,72,73,76,88,91], with most reports documenting high rejection rates [70,71,73,76,88,91]. Ipilimumab followed by pembrolizumab was associated with T-cell-mediated rejection, with graft survival in one patient but graft loss in another [88,91]. Alhamad et al. described a case of metastatic melanoma receiving the same regimen, resulting in antibody-mediated rejection with subsequent graft loss [76]. Additionally, in their series of six patients, Venkatachalam et al. observed that one patient receiving ipilimumab and pembrolizumab experienced both acute cellular and antibody-mediated rejection with graft loss [71]. Similarly, Zehou et al. and O’connell et al. observed graft rejection in their patients treated with ipilimumab followed by nivolumab for metastatic melanoma [70,73]. Interestingly, Delyon et al. reported no graft rejection in a patient receiving the same combination for metastatic melanoma [72]. A combination of pembrolizumab followed by ipilimumab and then nivolumab was reported in a 38-year-old patient with metastatic melanoma, who experienced no graft rejection [71].

##### Risk of Rejection and Immunosuppressive Regimens

Rejection events were more observed in patients receiving corticosteroids as monotherapy, with T-cell-mediated rejection being more common than antibody-mediated rejection. O’Connell et al. reported that among four patients receiving prednisolone monotherapy, three cases experienced graft rejection. However, all three achieved stable disease and no death was recorded [73]. Similarly, Delyon et al. reported a series of four patients, two of whom were on prednisone and developed graft rejection, with subsequent graft loss. Their cancer responses varied, including cases of progressive disease and partial response [72]. Additionally, other studies reported graft rejection on prednisolone monotherapy, with mixed outcomes regarding cancer response and overall survival [70,71,74,76,77,78,84,85,87].

Conversely, aggressive regimens combining corticosteroids with other immunosuppressants including CNI, mTOR inhibitors, and antimetabolites were associated with lower graft rejection rates. Zehou et al. reported a series of six patients with varying immunosuppressive regimens administered concomitantly with ICIs [70]. One patient received prednisolone monotherapy and subsequently experienced graft rejection; meanwhile, the remaining five cases were maintained on combination immunosuppressive regimens including mycophenolate mofetil (MMF), mTOR inhibitors (everolimus or sirolimus), and prednisolone. Specifically, two patients received (MMF, everolimus and prednisolone), one was on sirolimus and prednisolone, one on everolimus and prednisone, and another was treated with everolimus, azathioprine, and prednisolone. Notably, all patients, except the one on everolimus, azathioprine, and prednisolone, maintained their graft and had no rejection despite ICI therapy [70]. In the same manner, Venkatachalam et al. observed that in four patients receiving a combination of prednisolone and mTOR inhibitors, three cases had no graft rejection [71].

On the other hand, patients on other combination therapies including tacrolimus and MMF, sirolimus and MMF, still experienced graft rejection. Kumar et al. reported two cases on MMF, sirolimus, and/or prednisolone who developed a TCMR with subsequent graft loss [92]. Hanna et al. and Tan et al. each reported, respectively, that a patient on tacrolimus and MMF, and another on tacrolimus and prednisolone, both developed a TCMR [88,89]. Additionally, Lesouhaitier et al. described two cases receiving tacrolimus and MMF and experiencing graft rejection [69]. The observed findings suggest that despite aggressive immunosuppressive regimens, some patients were still at high risk of developing graft rejection after initiation of ICIs.

##### Cancer Response and Patient Survival

Out of the 53 reported patients discussed in this review, 57% had progressive disease, 15% had a complete response, 15% had a partial response, and 13% had stable disease following treatment with ICIs.

Cancer response to monotherapy with PD-1 inhibitors varied across the included studies. Of the patients experiencing progressive disease, the majority were on a PD-1 inhibitor. Venkatachalam et al. observed that, in a series of six patients, four patients receiving PD-1 inhibitors, either nivolumab or pembrolizumab, had progressive disease and among them, two patients died due to disease progression [71]. This finding is consistent with Lesouhaitier et al. who reported that four among seven patients on PD-1 inhibitors experienced disease progression, leading to death in three cases [69].

While a considerable proportion of patients had progressive disease, others demonstrated favorable outcomes. O’Connell et al. reported a series of five patients, in which three were treated with pembrolizumab, one with nivolumab, and one with a combination of ipilimumab and nivolumab. Out of cases receiving PD-1 inhibitors, three achieved stable disease, while only one experienced disease progression leading to death [73].

Other reports further support this finding, documenting cases with disease stabilization on nivolumab as monotherapy [80,84,86]. Additionally, several studies documented complete or partial cancer responses to PD-1 inhibitors, suggesting a variability in treatment outcomes [69,72,74,78,83,85,89,90,92,93,95,96].

On the other hand, CTLA-4 inhibitor, ipilimumab, whether used as monotherapy or in combination, was predominantly associated with disease progression. In a series of six patients, Zehou et al. documented that three out of four patients who received ipilimumab alone and one treated with ipilimumab followed by nivolumab experienced disease progression, ultimately leading to their death [70]. Notably, only one patient received ipilimumab monotherapy and achieved partial cancer response but later died from a cardiac event [70]. Other reports align with this finding with various outcomes regarding patient survival [69,72,73,76,77,91].

Overall, patients receiving monotherapy experienced higher mortality rates compared to combined therapies. Of all our included reports, only four reported deaths from cancer progression in patients treated with combination regimens [70,72,73,91]. Importantly, most deaths were not associated with graft rejection [70,71,72,73,77,79,82,87,91,94]. Similarly, Lesouhaitier et al. reported two patients who experienced graft rejection but died from disease progression [69].

Taken together, data from prospective trials, retrospective cohorts, and case series reveal two key patterns, although the absence of direct comparisons limits firm conclusions. First, immune checkpoint inhibitor therapy in kidney transplant recipients carries a substantial risk of acute rejection and treatment-related allograft loss, especially with dual checkpoint blockade. Second, maintaining or switching to a low-dose, multi-agent regimen that includes an mTOR inhibitor appears to lessen, though not eliminate, this risk. These trends closely parallel the findings of a recently published meta-analysis [97]. These observations highlight the need for carefully balanced immunosuppression and well-designed prospective studies to determine the safest strategy for patients who require both a functioning graft and effective cancer control.

## 5. Conclusions

While ICIs can offer significant antitumor benefits in patients with post-transplant malignancies, they also pose an increased risk of allograft rejection, primarily due to augmented immune activation. Clinicians must balance these immunologic trade-offs by carefully choosing their cancer treatment regimens, monitoring for signs of rejection, and integrating immunosuppressive regimens that minimize rejection risk without undermining ICI efficacy. This review touches on the potential of non-invasive biomarkers such as donor-derived cell-free DNA, for the early detection of rejection, as well as the role of tailored immunosuppression regimens (e.g., mTOR inhibitors instead of calcineurin inhibitors), which might enable the safer use of checkpoint blockade. Prospective head-to-head randomized trials are required before any modification of standard immunosuppressive protocols can be recommended. Collaboration between oncologists and transplant providers, close clinical surveillance, and rapid intervention for immune-mediated toxicities emerge as critical measures to ensure the safest possible outcomes. Overall, the literature highlights both the promise and the complexity of integrating immunotherapy into the standard care for transplant recipients facing difficult-to-treat cancers. Larger studies are warranted to gather further insights into the utilization of ICIs in KT recipients.

## Figures and Tables

**Table 1:** Summary of ICI adverse events.

Adverse Event	Clinical Presentation/Features
Allograft Rejection	Fever, fatigue, graft tenderness, elevated creatinine, proteinuria, hematuria [40,41,42]
Skin Toxicities	Rash, pruritus, vitiligo, severe forms include Stevens–Johnson syndrome [43,44]
Endocrinopathies	Hypothyroidism, transient hyperthyroidism, autoimmune diabetes mellitus [44,45,46]
Hepatotoxicity	Elevated liver enzymes, lobular hepatitis, autoimmune hepatitis-like patterns [47]
Gastrointestinal Disorders	Colitis, abdominal pain, mucosal hyperenhancement on imaging [48,49]
Pneumonitis	Cough, dyspnea, hypoxia, ground-glass opacities or interstitial patterns [50,51]
Acute Kidney Injury	Acute interstitial nephritis [52], elevated serum creatinine [53,54]
Rare Immune-Related Adverse Events	Autoimmune encephalitis [55], Guillain–Barré syndrome [56], myocarditis [57], autoimmune hemolytic anemia [58]

**Table 2:** Summary of immune checkpoint inhibitor studies in kidney transplant recipients.

Author	Study Design (*n*)	Age (Years) (IQR)	ICI	Dose	Cancer Treated	Time from Kidney Transplant to ICI Administration (IQR)	Immunosuppressive Regimen	Rejection	Graft Survival	Cancer Response	Mortality
Clinical Trials											
Schenk et al., 2024 [64]	Multicenter phase I/II trial (14)	(*n* = 8) Nivolumab group 66 (44–81)	Nivolumab	480 mg IV once every 4 weeks	5 patients with cSCC, 2 patients with Merkel cell carcinoma 1 patient with metastatic melanoma	13 years (8.7–21.4)	Tacrolimus and prednisolone	T-cell-mediated and antibody-mediated rejection in the metastatic melanoma patient	Yes, except for the metastatic melanoma patient	Progressive disease in all	Total deaths: 6, out of which 5 died within a year
		(*n* = 6) Nivolumab and ipilimumab 65 (44–77)	Nivolumab and Ipilimumab	Ipilimumab as 1 mg/kg and nivolumab as 3 mg/kg IV once every 3 weeks for 4 doses	3 patients with cSCC 2 patients with Merkel cell carcinoma 1 patient with metastatic melanoma	11.7 years (8.7–21.4)		T-cell-mediated and antibody-mediated rejection in 1 patient with Merkel cell carcinoma and T-cell-mediated rejection only in the patient with cSCC	Yes, except for 1 patient with Merkel cell carcinoma and 1 with cSCC	Progressive disease in the 2 patients with Merkel cell carcinoma and the patient with metastatic melanoma Stable disease in 1 patient with cSCC complete response in 2 patients with cSCC	
Hanna et al., 2024 [65]	Phase I, single-arm, single-center, non-randomized trial (12)	62.5 (43–86)	Cemiplimab	Cemiplimab at a dose of 350 mg every 21 days for up to 35 doses over 2 years	Advanced cSCC, Metastatic disease in 7 patients	7.2 years (2.8–21.1)	mTOR inhibitor and Prednisone 40 mg once, day before and the day of each cemiplimab cycle followed by 20 mg once daily on days 4–6, 10 mg once daily on day 7 continued till the day before each cycle	No	Yes	Three patients achieved a complete response (CR), two had a partial response (PR), and two patients exhibited stable disease	One patient died due to angioedema related to everolimus and an angiotensin-converting enzyme inhibitor Two deaths were attributed to progressive disease
Carroll RP et al., 2022 [66]	Prospective multicenter single-arm phase 1 trial (17)	67 (59–71)	Nivolumab	An infusion of nivolumab at 3 mg/kg every 2 weeks, for the first 5 doses After that, a fixed 480 mg infusion once every 4 weeks, continued for up to 2 years	6 patients with cSCC of the head and neck 3 patients with SCC of the head and neck and oropharynx 2 patients with renal tract carcinoma 2 patients with Merkel cell carcinoma 1 patient with Hepatocellular carcinoma (HCC) 1 patient with melanoma 1 patient with non-small cell lung cancer 1 patient with colorectal cancer	15.6 years (6.6–20.4)	Low-dose prednisone and Tacrolimus +/− MMF	Rejection occurred in 2 patients	1 graft loss; death-censored 2-year kidney allograft survival 89% (8/9)	Complete response: 4 (24%); partial response: 5 (29%)	9/17 deaths (all cancer-related)
Retrospective Cohort Studies											
Owoyemi et al., 2020 [67]	Retrospective cohort (7)	69 (53–70)	Nivolumab in 2 patients Pembrolizumab in 2 patients Cemiplimab in 2 patients Nivolumab followed by atezolizumab in 1 patient	NA	Metastatic cSCC in 4 patients NSCLC (adenocarcinoma) in 1 patient metastatic melanoma in 1 patient breast cancer in 1 patient	NA	Tacrolimus, MMF, and prednisone in 1 patient Tacrolimus and prednisone in 2 patients Sirolimus in 1 patient Sirolimus and prednisone in 2 patients prednisone only in 1 patient	1/7 (14%) Only the patient who received cemiplimab experienced rejection	Yes	Progressive disease in 4 patients stable disease in 3 patients	4/7 (57%) died (3 due to cancer progression, 1 due to infection after colitis)
Murakami et al., 2021 [68]	Retrospective cohort (69)	65 (55–71)	29 patients on pembrolizumab 11 patients on nivolumab 10 patients on cemiplimab 3 patients on atezolizumab 3 patients on avelumab 2 patients on ipilimumab 11 patients on PD-1/CTLA-4 combination	Standard FDA-labeled doses for each agent (2- or 3-week anti-PD-1/PD-L1; 3-week ipilimumab; Q3-weekly nivolumab + ipilimumab)	24 patients with metastatic cSCC 8 patients with NSCLC 4 patients with Merkel cell carcinoma 3 patients with renal cell carcinoma 2 patients with bladder cancer 6 patients with other cancers	9.33 years (4.1–15.6)	85% on steroids; 49% on 2-drug regimens; 55% on mTORi, 35% on CNI 65% had regimen changes immediately before ICI (most commonly CNI to mTOR, antimetabolite stopped, steroid increased)	29/69 (42%) developed rejection Biopsy proven rejections were 14 TCMR: 7 patients, Mixed TCMR and ABMR: 7	19/69 (28%) graft losses	Complete response in 5 patients partial response in 15 patients stable disease in 11 patients progressive disease in 34 patients unknown response in 4 patients	16 deaths among rejection cases
Case Series											
Lesouhaitier et al., 2018 [69]	Case series (7)	57	Nivolumab	5 doses	NSCLC (adenocarcinoma)	2.25 years	Steroid and mTOR inhibitor	No	Yes	Progressive disease	Yes
		70	Pembrolizumab	4 doses	Metastatic melanoma	8.75 years	Steroid and MMF	No	Yes	Complete response	No
		72	Avelumab	8 doses	Merckel cell carcinoma	3.5 years	Steroid and mTOR inhibitor	No	Yes	Progressive disease	Yes
		68	Ipilimumab	4 doses	Metastatic melanoma	0.75 years	Steroid, MMF, and mTOR inhibitor	No	Yes	Progressive disease	Yes
		64	Nivolumab	9 doses	NSCLC (adenocarcinoma)	6 years	Tacrolimus and MMF	Yes	No	Progressive disease	Yes
		73	Nivolumab	2 doses	Metastatic melanoma	1.25 years	Tacrolimus and MMF	Yes	No	Progressive disease	Yes
		85	Pembrolizumab	2 doses	Metastatic melanoma	28 years	Cyclosporine	Yes	Yes	Progressive disease	No
Zehou et al., 2018 [70]	Case series (6)	67	Ipilimumab	4 doses	Metastatic melanoma	2.25 years	MMF, everolimus, and prednisone 10 mg/day	No	Yes	Progressive disease	Death from tumor progression
		57	Ipilimumab	4 doses		5.5 years	Sirolimus and prednisone	No	Yes	Progressive disease	Death from tumor progression
		74	Ipilimumab then nivolumab	3 doses of ipilimumab then 1 dose of nivolumab		4.75 years	Everolimus, azathioprine, and prednisone 5 mg/day	No	Yes	Progressive disease	Death from tumor progression
		68	Ipilimumab	4 doses		0.8 years	MMF, everolimus, and prednisone 20 mg/day	No	Yes	Progressive disease	Death from tumor progression
		44	Ipilimumab then dacarbazine	1 dose of Ipilimumab, then 1 dose of dacarbazine		26 years	Prednisone 20 mg/day	T-cell-mediated rejection	Yes	Stable disease	Death from tumor progression and infection
		66	Ipilimumab	4 doses		23.5 years	Everolimus and prednisone 5 mg/day	No	Yes	Partial response	Death from cardiac disorder
Venkatachalam et al., 2019 [71]	Case series (6)	69	Pembrolizumab	NA	Metastatic cSCC	2 years	Prednisone 5 mg daily and everolimus 0.75 mg BID	T-cell-mediated rejection	No	Progressive disease	Yes
		67	Pembrolizumab	NA	Metastatic cSCC	22 years	Prednisone 7.5 mg daily and everolimus 0.5 mg BID	No	Yes	Progressive disease	Yes
		56	Nivolumab	NA	Renal cell carcinoma	2 years	Prednisone 5 mg daily and everolimus with target trough levels of 4–6 ng/ml	No	Yes	Progressive disease	NA
		38	Pembrolizumab then ipilimumab then nivolumab	NA	Metastatic melanoma	20 years	Sirolimus, and prednisone 10 mg daily, then maintained on prednisone 10 mg daily alone	No	Yes	Progressive disease	NA
		68	Ipilimumab then pembrolizumab	4 doses of ipilimumab then 1 dose of pembrolizumab	Metastatic melanoma	15 years	Prednisone 5 mg daily	Acute cellular and antibody-mediated rejection	No	Complete response	No
		58	Pembrolizumab	NA	NSCLC (adenocarcinoma)	10 years	Prednisone 10 mg daily	No	Yes	Progressive disease	NA
Delyon et al., 2020 [72]	Case series (4)	66	Cemiplimab	5 cycles, 3 mg/kg every 2 weeks	cSCC	24 years	Prednisone 10 mg/d	Yes	No	Progressive disease	Death from tumor progression
		63	Pembrolizumab	1 cycle, 2 mg/kg every 3 weeks	Kaposi sarcoma	9 years	Prednisone 7.5 mg/d	Yes	No	Partial response	No
		76	Avelumab	3 cycles, 10 mg/kg every 2 weeks	Merkel cell carcinoma	21 years	Cyclosporine and MMF	No	Yes	Progressive disease	Death from tumor progression
		55	Ipilimumab then nivolumab	1 cycle, Ipilimumab 3 mg/kg every 3 weeks nivolumab 1 mg/kg every 3 weeks	BRAF wild-type melanoma	2 years	Cyclosporine, Dexamethasone	No	Yes	Progressive disease	Death from tumor progression
O’Connell et al., 2025 [73]	Case series (5)	69	Pembrolizumab	NA	Metastatic melanoma	5 years	Prednisolone	No	Yes	Progressive disease	Yes
		66	Nivolumab		Metastatic melanoma	21 years	Prednisolone	Yes	No	Stable disease	No
		62	Pembrolizumab		Metastatic cSCC	12 years	Prednisolone	Yes	No	Stable disease	No
		59	Ipilimumab + nivolumab		Metastatic melanoma	1.5 years	Sirolimus and doubled dose of prednisolone	T-cell-mediated rejection	No	Progressive disease	Yes
		59	Pembrolizumab		Metastatic melanoma	22 years	Doubled dose of prednisolone	Yes	No	Stable disease	No
Case Reports											
Ong et al., 2016 [74]	Case report (1)	63	Nivolumab	A single intravenous dose of 324 mg	Metastatic melanoma	12 years	Prednisone 10 mg daily	T-cell-mediated rejection	No	Complete response	No
Herz et al., 2016 [75]	Case report (1)	77	Ipilimumab (initially), then Nivolumab	Ipilimumab: 3 mg/kg BW q3wk Nivolumab: 3 mg/kg BW q2wk	Metastatic melanoma	8 years	Prednisone 5 mg daily and tacrolimus 2 mg BID	No	Yes	Progressive disease	No
Alhamad et al., 2016 [76]	Case report (1)	68	Ipilimumab then Pembrolizumab	Ipilimumab as 3 mg/kg every 3 weeks / One dose of pembrolizumab before rejection	Metastatic melanoma	15 years	Prednisone 5 mg daily	Antibody-mediated rejection	No	Progressive disease	No
Jose et al., 2016 [77]	Case report (1)	40	Ipilimumab	2 cycles of Ipilimumab as 3 mg/kg every 3 weeks	Metastatic melanoma	16 years	Prednisone 5 mg daily	T-cell-mediated rejection	No	Progressive disease	Death from tumor progression
Lipson et al., 2016 [78]	Case report (1)	57	Pembrolizumab	NA	Metastatic cSCC	25 years	Prednisone 5 mg daily	T-cell-mediated rejection	No	Partial response	No
Kwatra et al., 2017 [79]	Case report (1)	58	Pembrolizumab	2 cycles of pembrolizumab 2 mg/kg every 3 weeks	Metastatic melanoma	16 years	Azathioprine 100 mg daily and everolimus 0.5 mg twice daily	T-cell-mediated rejection	No	Progressive disease	Yes, the patient refused the options of hemodialysis
Barnett et al., 2017 [80]	Case report (1)	70	Nivolumab	3 mg/kg intravenously every 2 weeks	Metastatic adenocarcinoma of the duodenum	6 years	Prednisone 20 mg daily and sirolimus 4–6 ng per milliliter	T-cell-mediated rejection	Yes	Stable disease	No
Winkler et al., 2017 [81]	Case report (2)	60	Nivolumab	NA	Metastatic melanoma	13 years	Prednisolone and MMF	No	Yes	Progressive disease	Death from tumor progression
		58	Pembrolizumab	NA		32 years	Cyclosporine			Progressive disease	Death from tumor progression
Deltombe et al., 2017 [82]	Case report (1)	73	Nivolumab	2 cycles of 3 mg/kg 30 days interval	Metastatic melanoma	15 months	Everolimus (2.5 mg/d)	T-cell-mediated rejection	No	Progressive disease	Death from tumor progression
Sadaat et al., 2017 [83]	Case report (1)	63	Pembrolizumab	4 cycles of 2 mg/kg every 3 weeks	Metastatic cSCC	13 years	Prednisone 2.5 mg and sirolimus 2 mg	No	Yes	Complete response	No
Goldman et al., 2018 [84]	Case report (1)	50	Nivolumab	3 mg/kg every 2 weeks	Metastatic cSCC	8.5 years	Prednisone 5 mg daily	T-cell-mediated rejection	No	Stable disease	No
Akturk et al., 2018 [85]	Case report (1)	52	Pembrolizumab (200 mg), then Nivolumab (240 mg)	IV infusion of 200 mg pembrolizumab followed by 240 mg of IV nivolumab in 2 weeks The patient continued nivolumab therapy, receiving eight additional infusions over 6 months	Metastatic melanoma	10 years	Prednisone 10 mg daily	T-cell-mediated rejection	No	Partial response	No
Singh et al., 2018 [86]	Case report (1)	71	Nivolumab	13 cycles of nivolumab (240 mg, 3 mg/kg per month)	Merkel cell carcinoma	12 years	Prednisone 10 mg daily	No	Yes	Stable disease with complete resolution of cancer symptoms	No
Hurkmans et al., 2019 [87]	Case report (1)	72	Nivolumab	4 doses of 3 mg/kg every 2 weeks	Metastatic melanoma	5 years	Prednisolone 20 mg/day	T-cell-mediated rejection	No	Progressive disease	Yes
Hanna et al., 2019 [88]	Case report (1)	52	Ipilimumab then pembrolizumab	8 cycles, dose NR	Metastatic melanoma	5 years	Tacrolimus 1.5 mg twice daily and MMF 250 mg twice daily and later Prednisone 20 mg daily	T-cell-mediated rejection	Yes	Excellent partial response	No
Tan et al., 2020 [89]	Case report (1)	71	Nivolumab	480 mg every 4 weeks	Metastatic melanoma	16 years	Tacrolimus 1.5 mg BID and prednisolone 5 mg daily	T-cell-mediated rejection	No	Complete response	No
Padala et al., 2020 [90]	Case report (1)	46	Pembrolizumab	NA	Metastatic endometrial adenocarcinoma	10 years	Sirolimus and prednisone	Yes	No	Partial response	No
Soellradl et al., 2020 [91]	Case report (1)	72	Ipilimumab then pembrolizumab	4 cycles of ipilimumab 1 cycle of pembrolizumab	Metastatic melanoma	8 years	Sirolimus	T-cell-mediated rejection	No	Progressive disease	Death from tumor progression and severe candida sepsis
Kumar et al., 2024 [92]	Case report (2)	66	Pembrolizumab	11 cycles, 200 mg intravenous every 3 weeks	Metastatic cSCC	14.5 years	MMF 500 BID, sirolimus 2 mg daily, and prednisolone 20 mg daily	T-cell-mediated rejection	Yes	Complete response	No
		78	Pembrolizumab	2 cycles, 200 mg intravenous every 3 weeks	Metastatic melanoma	12 years	MMF 750 mg BID and sirolimus 1 mg daily and prednisone 5 mg daily	T-cell-mediated rejection		Partial response	
Paoluzzi et al., 2021 [93]	Case report (1)	72	Cemiplimab	10 cycles, 350 mg IV every 3 weeks	Locally advanced cSCC	10 years	Prednisone 5 mg/d	No	Yes	Partial response	No
Ishikawa et al., 2021 [94]	Case report (1)	64	Nivolumab	3 doses of nivolumab 3 mg/kg every 2 weeks	metastatic RCC	9 years	NA	T-cell-mediated rejection	Yes	Progressive disease	Death from tumor progression
Lu et al., 2023 [95]	Case report (1)	74	Pembrolizumab	4 cycles of pembrolizumab	Metastatic cSCC	6 years	Everolimus and prednisone 5 mg daily	No	Yes	Complete response	No
Antonelli et al., 2024 [96]	Case report (1)	Early 40 s	Pembrolizumab	19 cycles of 200 mg intravenous every 3 weeks	Metastatic cSCC	9 years	Sirolimus (goal of 4–8 ng/mL) and prednisone 5 mg twice daily	No	Yes	Complete response	No

ABMR: antibody-mediated rejection; BID: twice daily; BW: body weight; CNI: calcineurin inhibitor; CR: complete response; cSCC: cutaneous squamous cell carcinoma; CTLA-4: cytotoxic T-lymphocyte-associated protein 4; FDA: Food and Drug Administration; HCC: hepatocellular carcinoma; ICI: immune checkpoint inhibitor; IQR: interquartile range; IV: intravenous; MMF: mycophenolate mofetil; mTORi: mammalian target of rapamycin inhibitor; NA: not available; NE: not estimable; NSCLC: non-small cell lung cancer; PD: progressive disease; PD-1: programmed death 1; PD-L1: programmed death ligand 1; PR: partial response; RCC: renal cell carcinoma; SD: stable disease; SCC: squamous cell carcinoma; TCMR: T-cell-mediated rejection; TRAL: treatment-related allograft loss.

## References

[1] Tonelli M., Wiebe N., Knoll G., Bello A., Browne S., Jadhav D., Klarenbach S., Gill J. (2011). Systematic review: Kidney transplantation compared with dialysis in cl inically relevant outcomes. Am. J. Transplant..

[2] Pesavento T.E. (2009). Kidney Transplantation in the Context of Renal Replacement Therapy. Clin. J. Am. Soc. Nephrol..

[3] Agrawal A., Ison M.G., Danziger-Isakov L. (2022). Long-Term Infectious Complications of Kidney Transplantation. Clin. J. Am. Soc. Nephrol..

[4] Gaston R.S. (2016). Improving long-term outcomes in kidney transplantation: Towards a new paradigm of post-transplant care in the united states. Trans. Am. Clin. Climatol. Assoc..

[5] Joosten S.A., Sijpkens Y.W., Van Kooten C., Paul L.C. (2005). Chronic renal allograft rejection: Pathophysiologic considerations. Kidney Int..

[6] Kasiske B.L., Snyder J.J., Gilbertson D.T., Wang C. (2004). Cancer after kidney transplantation in the United States. Am. J. Transplant..

[7] Collett D., Mumford L., Banner N.R., Neuberger J., Watson C. (2010). Comparison of the incidence of malignancy in recipients of different t ypes of organ: A UK Registry audit. Am. J. Transplant..

[8] Cheung C.Y., Tang S.C.W. (2019). An update on cancer after kidney transplantation. Nephrol. Dial. Transplant..

[9] Al-Adra D., Al-Qaoud T., Fowler K., Wong G. (2022). De Novo Malignancies after Kidney Transplantation. Clin. J. Am. Soc. Nephrol..

[10] Ietto G., Gritti M., Pettinato G., Carcano G., Gasperina D.D. (2023). Tumors after kidney transplantation: A population study. World J. Surg. Oncol..

[11] Mittal A., Colegio O.R. (2017). Skin Cancers in Organ Transplant Recipients. Am. J. Transplant..

[12] Gioco R., Corona D., Agodi A., Privitera F., Barchitta M., Giaquinta A., Alba I., D’Errico S., Pinto F., De Pasquale C. (2019). De Novo Cancer Incidence and Prognosis After Kidney Transplantation: A Single Center Analysis. Transplant. Proc..

[13] Opelz G., Döhler B. (2004). Lymphomas after solid organ transplantation: A collaborative transplan t study report. Am. J. Transplant..

[14] Smyth M.J., Godfrey D.I., Trapani J.A. (2001). A fresh look at tumor immunosurveillance and immunotherapy. Nat. Immunol..

[15] Bromberg J.F., Horvath C.M., Wen Z., Schreiber R.D., Darnell J.E. (1996). Transcriptionally active Stat1 is required for the antiproliferative effects of both interferon alpha and interferon gamma. Proc. Natl. Acad. Sci. USA.

[16] Coughlin C.M., Salhany K.E., Gee M.S., LaTemple D.C., Kotenko S., Ma X., Gri G., Wysocka M., Kim J.E., Liu L. (1998). Tumor cell responses to IFNgamma affect tumorigenicity and response to IL-12 therapy and antiangiogenesis. Immunity.

[17] Gerosa F., Baldani-Guerra B., Nisii C., Marchesini V., Carra G., Trinchieri G. (2002). Reciprocal activating interaction between natural killer cells and dendritic cells. J. Exp. Med..

[18] Piccioli D., Sbrana S., Melandri E., Valiante N.M. (2002). Contact-dependent stimulation and inhibition of dendritic cells by natural killer cells. J. Exp. Med..

[19] Dunn G.P., Bruce A.T., Ikeda H., Old L.J., Schreiber R.D. (2002). Cancer immunoediting: From immunosurveillance to tumor escape. Nat. Immunol..

[20] Piselli P., Busnach G., Fratino L., Citterio F., Ettorre G.M., De Paoli P., Serraino The Immunosuppression and Cancer Study Group, D. (2013). De novo malignancies after organ transplantation: Focus on viral infections. Curr. Mol. Med..

[21] Rostaing L., Wéclawiak H., Mengelle C., Kamar N. (2011). Viral infections after kidney transplantation. Ital. J. Urol. Nephrol..

[22] Elkhalifa A.M.E., Nabi S.U., Shah O.S., Bashir S.M., Muzaffer U., Ali S.I., Wani I.A., Alzerwi N.A.N., Elderdery A.Y., Alanazi A. (2023). Insight into Oncogenic Viral Pathways as Drivers of Viral Cancers: Implication for Effective Therapy. Curr. Oncol..

[23] Pierangeli A., Antonelli G., Gentile G. (2015). Immunodeficiency-associated viral oncogenesis. Clin. Microbiol. Infect..

[24] Tower H., Ruppert M., Britt K. (2019). The Immune Microenvironment of Breast Cancer Progression. Cancers.

[25] Thompson J.A. (2018). New NCCN Guidelines: Recognition and Management of Immunotherapy-Relat ed Toxicity. J. Natl. Compr. Cancer Netw. JNCCN.

[26] Cui X., Yan C., Xu Y., Li D., Guo M., Sun L., Zhu Z. (2023). Allograft rejection following immune checkpoint inhibitors in solid or gan transplant recipients: A safety analysis from a literature review and a pharmacovigilance system. Cancer Med..

[27] Nguyen L.S., Ortuno S., Lebrun-Vignes B., Johnson D.B., Moslehi J.J., Hertig A., Salem J.-E. (2021). Transplant rejections associated with immune checkpoint inhibitors: A pharmacovigilance study and systematic literature review. Eur. J. Cancer.

[28] Babamohamadi M., Mohammadi N., Faryadi E., Haddadi M., Merati A., Ghobadinezhad F., Amirian R., Izadi Z., Hadjati J. (2024). Anti-CTLA-4 nanobody as a promising approach in cancer immunotherapy. Cell Death Dis..

[29] Meng L., Wu H., Wu J., Ding P.a., He J., Sang M., Liu L. (2024). Mechanisms of immune checkpoint inhibitors: Insights into the regulation of circular RNAS involved in cancer hallmarks. Cell Death Dis..

[30] Gardner D., Jeffery L.E., Sansom D.M. (2014). Understanding the CD28/CTLA-4 (CD152) Pathway and Its Implications for Costimulatory Blockade. Am. J. Transplant..

[31] Walker L.S., Sansom D.M. (2011). The emerging role of CTLA4 as a cell-extrinsic regulator of T cell res ponses. Nat. Rev. Immunol..

[32] Schwartz R.H. (1990). A cell culture model for T lymphocyte clonal anergy. Science.

[33] Francisco L.M., Salinas V.H., Brown K.E., Vanguri V.K., Freeman G.J., Kuchroo V.K., Sharpe A.H. (2009). PD-L1 regulates the development, maintenance, and function of induced regulatory T cells. J. Exp. Med..

[34] Kooshkaki O., Derakhshani A., Hosseinkhani N., Torabi M., Safaei S., Brunetti O., Racanelli V., Silvestris N., Baradaran B. (2020). Combination of Ipilimumab and Nivolumab in Cancers: From Clinical Practice to Ongoing Clinical Trials. Int. J. Mol. Sci..

[35] Kalia V., Penny L.A., Yuzefpolskiy Y., Baumann F.M., Sarkar S. (2015). Quiescence of Memory CD8^+^ T Cells Is Mediated by Regulatory T Cells through Inhibitory Receptor CTLA-4. Immunity.

[36] Førde D., Kilvær T., Pedersen M.I., Blix E.S., Urbarova I., Paulsen E.-E., Rakaee M., Busund L.-T.R., Donnem T., Andersen S. (2024). High density of TCF1+ stem-like tumor-infiltrating lymphocytes is associated with favorable disease-specific survival in NSCLC. Front. Immunol..

[37] Ouyang P., Wang L., Wu J., Tian Y., Chen C., Li D., Yao Z., Chen R., Xiang G., Gong J. (2024). Overcoming cold tumors: A combination strategy of immune checkpoint inhibitors. Front. Immunol..

[38] Wang L., Geng H., Liu Y., Liu L., Chen Y., Wu F., Liu Z., Ling S., Wang Y., Zhou L. (2023). Hot and cold tumors: Immunological features and the therapeutic strategies. MedComm.

[39] Liu Y.T., Sun Z.J. (2021). Turning cold tumors into hot tumors by improving T-cell infiltration. Theranostics.

[40] Morrison S.A., Vinson A.J. (2024). Acute Allograft Rejection in Kidney Transplant Recipients Treated With Immune Checkpoint Inhibitors: An Educational Case Report. Can. J. Kidney Health Dis..

[41] Goldberg R.J., Weng F.L., Kandula P. (2016). Acute and Chronic Allograft Dysfunction in Kidney Transplant Recipients. Med. Clin. N. Am..

[42] Aguirre L.E., Guzman M.E., Lopes G., Hurley J. (2019). Immune Checkpoint Inhibitors and the Risk of Allograft Rejection: A Co mprehensive Analysis on an Emerging Issue. Oncologist.

[43] Belum V.R., Benhuri B., Postow M.A., Hellmann M.D., Lesokhin A.M., Segal N.H., Motzer R.J., Wu S., Busam K.J., Wolchok J.D. (2016). Characterisation and management of dermatologic adverse events to agen ts targeting the PD-1 receptor. Eur. J. Cancer.

[44] Haanen J.B.A.G., Carbonnel F., Robert C., Kerr K.M., Peters S., Larkin J., Jordan K. (2017). Management of toxicities from immunotherapy: ESMO Clinical Practice Gu idelines for diagnosis, treatment and follow-up. Ann. Oncol..

[45] Osorio J.C., Ni A., Chaft J.E., Pollina R., Kasler M.K., Stephens D., Rodriguez C., Cambridge L., Rizvi H., Wolchok J.D. (2017). Antibody-mediated thyroid dysfunction during T-cell checkpoint blockad e in patients with non-small-cell lung cancer. Ann. Oncol..

[46] Karaviti D., Kani E.R., Karaviti E., Gerontiti E., Michalopoulou O., Stefanaki K., Kazakou P., Vasileiou V., Psaltopoulou T., Paschou S.A. (2024). Thyroid disorders induced by immune checkpoint inhibitors. Endocrine.

[47] Da Cunha T., Wu G.Y., Vaziri H. (2022). Immunotherapy-induced Hepatotoxicity: A Review. J. Clin. Transl. Hepatol..

[48] Losurdo G., Angelillo D., Favia N., Sergi M.C., Di Leo A., Triggiano G., Tucci M. (2023). Checkpoint Inhibitor-Induced Colitis: An Update. Biomedicines.

[49] Mooradian M.J., Wang D.Y., Coromilas A., Lumish M., Chen T., Giobbie-Hurder A., Johnson D.B., Sullivan R.J., Dougan M. (2020). Mucosal inflammation predicts response to systemic steroids in immune checkpoint inhibitor colitis. J. Immunother. Cancer.

[50] Nishino M., Sholl L.M., Hatabu H., Ramaiya N.H., Hodi F.S. (2015). Anti–PD-1–Related Pneumonitis during Cancer Immunotherapy. N. Engl. J. Med..

[51] Khunger M., Rakshit S., Pasupuleti V., Hernandez A.V., Mazzone P., Stevenson J., Pennell N.A., Velcheti V. (2017). Incidence of Pneumonitis With Use of Programmed Death 1 and Programmed Death-Ligand 1 Inhibitors in Non-Small Cell Lung Cancer: A Systematic Review and Meta-Analysis of Trials. Chest.

[52] Murakami N., Motwani S., Riella L.V. (2017). Renal complications of immune checkpoint blockade. Curr. Probl. Cancer.

[53] Gupta S., Short S.A.P., Sise M.E., Prosek J.M., Madhavan S.M., Soler M.J., Ostermann M., Herrmann S.M., Abudayyeh A., Anand S. (2021). Acute kidney injury in patients treated with immune checkpoint inhibit ors. J. Immunother. Cancer.

[54] Cortazar F.B., Kibbelaar Z.A., Glezerman I.G., Abudayyeh A., Mamlouk O., Motwani S.S., Murakami N., Herrmann S.M., Manohar S., Shirali A.C. (2020). Clinical Features and Outcomes of Immune Checkpoint Inhibitor-Associat ed AKI: A Multicenter Study. J. Am. Soc. Nephrol..

[55] Gao Y., Pan J., Shen D., Peng L., Mao Z., Wang C., Meng H., Zhou Q., Chen S. (2022). Immune Checkpoint Inhibitor Associated Autoimmune Encephalitis, Rare and Novel Topic of Neuroimmunology: A Case Report and Review of the Literature. Brain Sci..

[56] Marini A., Bernardini A., Gigli G.L., Valente M., Muñiz-Castrillo S., Honnorat J., Vogrig A. (2021). Neurologic Adverse Events of Immune Checkpoint Inhibitors. Neurology.

[57] Palaskas N., Lopez-Mattei J., Durand J.B., Iliescu C., Deswal A. (2020). Immune Checkpoint Inhibitor Myocarditis: Pathophysiological Characteristics, Diagnosis, and Treatment. J. Am. Heart Assoc..

[58] Hwang S.R., Saliba A.N., Wolanskyj-Spinner A.P. (2022). Immunotherapy-associated Autoimmune Hemolytic Anemia. Hematol./Oncol. Clin. N. Am..

[59] Kitchlu A., Jhaveri K.D., Wadhwani S., Deshpande P., Harel Z., Kishibe T., Henriksen K., Wanchoo R. (2021). A Systematic Review of Immune Checkpoint Inhibitor-Associated Glomerular Disease. Kidney Int. Rep..

[60] Herrmann S.M., Abudayyeh A., Gupta S., Gudsoorkar P., Klomjit N., Motwani S.S., Karam S., Costa E.S.V.T., Khalid S.B., Anand S. (2025). Diagnosis and management of immune checkpoint inhibitor-associated nephrotoxicity: A position statement from the American Society of Onco-nephrology. Kidney Int..

[61] Gumusay O., Callan J., Rugo H.S. (2022). Immunotherapy toxicity: Identification and management. Breast Cancer Res. Treat..

[62] Perazella M.A., Shirali A.C. (2020). Immune checkpoint inhibitor nephrotoxicity: What do we know and what s hould we do?. Kidney Int..

[63] Mauiyyedi S., Crespo M., Collins A.B., Schneeberger E.E., Pascual M.A., Saidman S.L., Tolkoff-Rubin N.E., Williams W.W., Delmonico F.L., Cosimi A.B. (2002). Acute humoral rejection in kidney transplantation: II. Morphology, imm unopathology, and pathologic classification. J. Am. Soc. Nephrol..

[64] Schenk K.M., Deutsch J.S., Chandra S., Davar D., Eroglu Z., Khushalani N.I., Luke J.J., Ott P.A., Sosman J.A., Aggarwal V. (2024). Nivolumab + Tacrolimus + Prednisone ± Ipilimumab for Kidney Transplant Recipients With Advanced Cutaneous Cancers. J. Clin. Oncol..

[65] Hanna G.J., Dharanesswaran H., Giobbie-Hurder A., Harran J.J., Liao Z., Pai L., Tchekmedyian V., Ruiz E.S., Waldman A.H., Schmults C.D. (2024). Cemiplimab for Kidney Transplant Recipients With Advanced Cutaneous Squamous Cell Carcinoma. J. Clin. Oncol..

[66] Carroll R.P., Boyer M., Gebski V., Hockley B., Johnston J.K., Kireta S., Tan H., Taylor A., Wyburn K., Rzalcberg J. (2022). Immune checkpoint inhibitors in kidney transplant recipients: A multicentre, single-arm, phase 1 study. Lancet Oncol..

[67] Owoyemi I., Vaughan L.E., Costello C.M., Thongprayoon C., Markovic S.N., Herrmann J., Otley C.C., Taner T., Mangold A.R., Leung N. (2020). Clinical outcomes of solid organ transplant recipients with metastatic cancers who are treated with immune checkpoint inhibitors: A single-center analysis. Cancer.

[68] Murakami N., Mulvaney P., Danesh M., Abudayyeh A., Diab A., Abdel-Wahab N., Abdelrahim M., Khairallah P., Shirazian S., Kukla A. (2021). A multi-center study on safety and efficacy of immune checkpoint inhib itors in cancer patients with kidney transplant. Kidney Int..

[69] Lesouhaitier M., Dudreuilh C., Tamain M., Kanaan N., Bailly E., Legoupil D., Deltombe C., Perrin P., Manson G., Vigneau C. (2018). Checkpoint blockade after kidney transplantation. Eur. J. Cancer.

[70] Zehou O., Leibler C., Arnault J.P., Sayegh J., Montaudie H., Remy P., Glotz D., Cordonnier C., Martin L., Lebbe C. (2018). Ipilimumab for the treatment of advanced melanoma in six kidney transplant patients. Am. J. Transplant..

[71] Venkatachalam K., Malone A.F., Heady B., Santos R.D., Alhamad T. (2020). Poor Outcomes With the Use of Checkpoint Inhibitors in Kidney Transplant Recipients. Transplantation.

[72] Delyon J., Zuber J., Dorent R., Poujol-Robert A., Peraldi M.-N., Anglicheau D., Lebbe C. (2021). Immune Checkpoint Inhibitors in Transplantation—A Case Series and Comp rehensive Review of Current Knowledge. Transplantation.

[73] O’Connell B., Cowhig C., McAnallen S., Hanko J.B., Naidoo J., Clarkson M.R., Conlon P.J. (2025). Immune Checkpoint Inhibitor Use in Kidney Transplant Patients: A National Case Series From Ireland. Clin. Transplant..

[74] Ong M., Ibrahim A.M., Bourassa-Blanchette S., Canil C., Fairhead T., Knoll G. (2016). Antitumor activity of nivolumab on hemodialysis after renal allograft rejection. J. Immunother. Cancer.

[75] Herz S., Hofer T., Papapanagiotou M., Leyh J.C., Meyenburg S., Schadendorf D., Ugurel S., Roesch A., Livingstone E., Schilling B. (2016). Checkpoint inhibitors in chronic kidney failure and an organ transplant recipient. Eur. J. Cancer.

[76] Alhamad T., Venkatachalam K., Linette G.P., Brennan D.C. (2016). Checkpoint Inhibitors in Kidney Transplant Recipients and the Potential Risk of Rejection. Am. J. Transplant..

[77] Jose A., Yiannoullou P., Bhutani S., Denley H., Morton M., Picton M., Summers A., van Dellen D., Augustine T. (2016). Renal Allograft Failure After Ipilimumab Therapy for Metastatic Melanoma: A Case Report and Review of the Literature. Transplant. Proc..

[78] Lipson E.J., Bagnasco S.M., Moore J., Jang S., Patel M.J., Zachary A.A., Pardoll D.M., Taube J.M., Drake C.G. (2016). Tumor Regression and Allograft Rejection after Administration of Anti-PD-1. N. Engl. J. Med..

[79] Kwatra V., Karanth N., Priyadarshana K., Charakidis M. (2016). Pembrolizumab for metastatic melanoma in renal allograft recipient with subsequent graft rejection and treatment response failure, a case report. Asia-Pac. J. Clin. Oncol..

[80] Barnett R., Barta V.S., Jhaveri K.D. (2017). Preserved Renal-Allograft Function and the PD-1 Pathway Inhibitor Nivolumab. N. Engl. J. Med..

[81] Winkler J.K., Gutzmer R., Bender C., Lang N., Zeier M., Enk A.H., Hassel J.C. (2017). Safe Administration of An Anti-PD-1 Antibody to Kidney-transplant Patients: 2 Clinical Cases and Review of the Literature. J. Immunother..

[82] Deltombe C., Garandeau C., Quereux G., Renaudin K., Hourmant M. (2017). Severe allograft rejection and autoimmune hemolytic anemia after anti-PD1 therapy in a kidney transplanted patient. Transpl. Int..

[83] Sadaat M., Jang S. (2018). Complete Tumor Response to Pembrolizumab and Allograft Preservation in Renal Allograft Recipient on Immunosuppressive Therapy. J. Oncol. Pract..

[84] Goldman J.W., Abdalla B., Mendenhall M.A., Sisk A., Hunt J., Danovitch G.M., Lum E.L. (2018). PD 1 checkpoint inhibition in solid organ transplants: 2 sides of a coin—Case report. BMC Nephrol..

[85] Akturk H.K., Alkanani A., Zhao Z., Yu L., Michels A.W. (2018). PD-1 inhibitor immune-related adverse events in patients with preexisting endocrine autoimmunity. J. Clin. Endocrinol. Metab..

[86] Singh P., Visger Von J., Prosek J., Rovin B., Pesavento T.E., Olencki T., Pandey D. (2019). Preserved Renal Allograft Function and Successful Treatment of Metastatic Merkel Cell Cancer Post Nivolumab Therapy. Transplantation.

[87] Hurkmans D.P., Verhoeven J., de Leur K., Boer K., Joosse A., Baan C.C., von Der Thüsen J.H., van Schaik R.H.N., Mathijssen R.H.J., van Der Veldt A.A.M. (2019). Donor-derived cell-free DNA detects kidney transplant rejection during nivolumab treatment. J. Immunother. Cancer.

[88] Hanna D.L., Law S.J., Merrick S.A., Heptinstall L., Bass P., Dupont P., Sheri A. (2020). The successful use of pembrolizumab in a renal transplant recipient with metastatic melanoma. Melanoma Res..

[89] Tan B., Baxter M., Casasola R. (2021). Acute renal transplant rejection following nivolumab therapy for metastatic melanoma. BMJ Case Rep..

[90] Padala S.A., Patel S.K., Vakiti A., Patel N., Gani I., Kapoor R., Muhammad S. (2021). Pembrolizumab-induced severe rejection and graft intolerance syndrome resulting in renal allograft nephrectomy. J. Oncol. Pharm. Pract..

[91] Soellradl I., Kehrer H., Cejka D. (2020). Use of Ipilimumab and Pembrolizumab in Metastatic Melanoma in a Combined Heart and Kidney Transplant Recipient: A Case Report. Transplant. Proc..

[92] Kumar V., Shinagare A.B., Rennke H.G., Ghai S., Lorch J.H., Ott P.A., Rahma O.E. (2020). The Safety and Efficacy of Checkpoint Inhibitors in Transplant Recipie nts: A Case Series and Systematic Review of Literature. Oncologist.

[93] Paoluzzi L., Ow T.J. (2021). Safe Administration of Cemiplimab to a Kidney Transplant Patient with Locally Advanced Squamous Cell Carcinoma of the Scalp. Curr. Oncol..

[94] Ishikawa G., Sugiyama T., Ito T., Otsuka A., Miyake H. (2021). Renal allograft rejection after treatment with nivolumab in patients with metastatic renal cell carcinoma. Int. Cancer Conf. J..

[95] Lu Z., Afzal M., Shirai K. (2023). Durable complete response to early immunotherapy discontinuation in a kidney transplant recipient with advanced cutaneous squamous cell carcinoma: A case report and review of literature. Transplant. Immunol..

[96] Antonelli J.P., Quach M., Mahajan A., Pleva J., Ma V.T. (2024). Rapid Life-Saving Response to Anti-PD-1 in a Solid Organ Transplant Recipient with Metastatic Cutaneous Squamous Cell Carcinoma: A Case Report and Review. J. Immunother..

[97] (2025). Saleem N, Wang J, Rejuso A; et al. Outcomes of Solid Organ Transplant Recipients With Advanced Cancers Receiving Immune Checkpoint Inhibitors: A Systematic Review and Individual Participant Data Meta-Analysis. JAMA Oncol..

